# A Proposal for Terminology for Clear Communication and Avoidance of Confusion in Relation to Sex, the Social Expectations of the Sexes and Gender Identity

**DOI:** 10.1007/s10508-025-03310-3

**Published:** 2025-10-29

**Authors:** Karleen D. Gribble

**Affiliations:** School of Nursing and Midwifery, Western Sydney University, Locked Bag 1797, Penrith, NSW 2751 Australia

Globally, an increased cultural salience of the concept of gender identity has stimulated rapid changes in law, policy, data collection, and communication (Bartick et al., 2025; Biggs, 2022; Gribble et al., 2022; Holzer, 2022; Munzer et al., 2025; Sullivan et al., 2025). These changes have the effect of marginalizing sex as a category of importance and have been noted as having a disproportionate negative impact on girls and women (Floris et al., 2018; Munzer et al., 2025; Murray & Hunter Blackburn, 2019; Sullivan, 2020). However, discussion regarding these changes and their implications has been hampered by use of the term “gender” to refer to the disparate concepts of sex, the social expectations of the sexes, and gender identity. This commentary outlines how “gender” has been applied to these different concepts and will propose alternative terminology to promote more effective discussion.

## Defining Sex, the Social Expectations of the Sexes and Gender Identity

Sex is a reproductive categorization determined by the two distinct developmental pathways toward the production of large sessile gametes (female) or small motile gametes (male) (Goymann et al., 2023; Hilton & Wright, 2023; Kodric-Brown & Brown, 1987). The reproductive strategy involving the fusion of a small gamete with a large gamete to produce a new individual is applied by all sexually reproducing animal species and is termed anisogamy (Subramoniam, 2017; Yasui & Hasegawa, 2022). The fact of just two sexes is a reflection of the existence of just two gamete types (Lehtonen & Parker, 2014).

Sex is related to but distinct from sex characteristics. Sex characteristics are features that differ between males and females, may be related to morphology, behavior, hormone production, or genetics, and are species-specific (Goymann et al., 2023). However, particularly in relation to humans, sex and sex characteristics are commonly conflated and confused (Fausto-Sterling, 1993; Goymann et al., 2023). Definitions of sex that refer to “chromosomal sex,” “anatomical sex,” “hormonal sex,” or “sex as a spectrum” (e.g., Ainsworth, 2015; Fausto-Sterling, 1993) are confusing sex with sex characteristics. Such confusion demonstrates a lack of understanding of sex as a cross-species categorization in which sex determination, sexual development, and sex characteristics vary (Goymann et al., 2023). For example, while sex in mammals is determined genetically, this is not the case for all species, and sex may also be determined by temperature (e.g., crocodiles and most turtles) or social factors (e.g., some fish) (Bachtrog et al., 2014).

Furthermore, defining sex in relation to sex characteristics allows for the suggestion that people who alter their secondary sex characteristics by medical means may have changed their sex. This risks negative ramifications for their individual health (e.g., Lao & Crawley, 2020; Stroumsa et al., 2019; Whitley & Greene, 2017) as well as the integrity of health and other data sets (Gribble et al., 2025). While some animal species (mostly invertebrates) are sequential hermaphrodites and capable of consecutively producing small and large gametes and so changing sex, no mammals can do this (Avise & Mank, 2009; Goymann et al., 2023). Thus, in humans, while some sex characteristics can be altered via medical means and some jurisdictions allow individuals to change their sex for some legal purposes, sex in humans is immutable (Goymann et al., 2023).

The social expectations of the sexes are the cultural meanings and expectations placed on people dependent upon whether they are male or female; that is, a girl, a woman, a boy, or a man (Wood & Eagly, 2012). These expectations come with rewards and sanctions for acting or not acting in line with them and in this way contribute to the development of socially constructed roles for men and women, to sex stereotyping, and to sexism (Wood & Eagly, 2012). Feminists have argued that the different expectations placed on women and men can and should be made irrelevant by political and social action (Mikkola, 2024). However, the difference between the bodies of male and female people is substantially explained by the physical requirements of gestating, birthing, and breastfeeding infants for women and the lack of such ability for men (Lassek & Gaulin, 2022). In turn, the differential reproductive abilities and associated biological and physical differences between men and women shape the expectations societies place on each (Wood & Eagly, 2012). This has been described as the “cultural interpretation of what is biologically given” (Gezen, 2020, p. 93). As a result, the sex-related and social and cultural factors underlying the social expectations of the sexes can be entwined and are often difficult to disentangle.

While sex and the social expectations of the sexes are as old as humans (in fact, older when it comes to sex), the concept of gender identity is new. The term “gender identity” was first used in the 1960s to describe a person’s knowledge and understanding of their sex as male or female (Byrne, 2023a). However, the meaning soon changed and became enmeshed with the social expectations of the sexes to delineate an internal sense of oneself as living in the social role of a man or a woman (Money, 1994). Over time, the concept of gender identity has further evolved, and while there is not a universally accepted meaning, and definitions tend to be circular, they also commonly refer what appears to be the social expectations of the sexes and sex stereotypes. For example, Australian law describes gender identity as “the gender-related identity, appearance or mannerisms or other gender-related characteristics of a person” (Australian Human Rights Commission, 2013, p. 3). The Sex and Gender Equity in Research (SAGER) guidelines define gender identity as “a person’s concept of self as being male and masculine or female and feminine, or ambivalent, based in part on physical characteristics, parental responses and psychological and social pressures. It is the internal experience of gender role” (Heidari et al., 2016, p. 7). In practice, sex stereotypes are prominent in considerations of gender identity. For example, Fausto-Sterling’s (2025) recent analysis of gender identity development in young children appears to be entirely focused on sex stereotypes, including children’s attraction to “boy toys” or “girl toys.”

During the twenty-first century, the concept of gender identity has become increasingly important in many contexts, entering into law in many countries and central to a number of United Nations resolutions (Murray et al., 2020; United Nations Human Rights Council, 2022). There are also an increasing number of individuals, particularly in Western nations, who report experiencing a gender identity in conflict with their sex, a state described as being transgender (McKechnie et al., 2023; Wiepjes et al., 2018; Zhang et al., 2021a). Such individuals may be male but say they have a gender identity of a woman, may be female and have a gender identity of a man, or they may have a gender identity of neither man nor woman or of both simultaneously (Galupo et al., 2017). Those with a transgender experience may place great importance on their gender identity and find it distressing to have their sex referred to (Jacobsen et al., 2024).

While it is often implied or explicitly stated that everyone has a gender identity (Byrne, 2023b), this claim is not evidenced and some people overtly reject applying gender identity to themselves, viewing it as an unhelpful or regressive concept (Gribble et al., 2023; Munzer et al., 2025). It is also unclear what the broader population understands gender identity to be, but there is reason to believe there may be many who are confused by the term (Biggs, 2024a, 2024b). The absence of an accepted and stable definition and the often-circular nature of definitions do not provide for clarity. Furthermore, conflation and confusion of gender identity with sex are encouraged by the common use of “gender” as a synonym for sex and representation of the scientific terms for the sexes, “male” and “female,” as gender identities (Gribble et al., 2025).

## The Multiple Meanings of “Gender”

The word “gender” has existed for centuries. For most of this time, it was overwhelmingly used to describe a grammatical feature of noun classification present in some languages (Audring, 2016; Oxford English Dictionary, 2024). As illustrative, Haig (2004) reported just two articles in Science Citation Index included “gender” in their title in a non-grammatical sense over the years 1945–1959. However, from around the 1970s, “gender” began to be widely used as a synonym for sex (Haig, 2004; Oxford English Dictionary, 2024). The preference for gender in this meaning seemingly came from sex increasingly being used to denote sexual intercourse and a desire not to connotate sexual behavior or associated concepts in contexts where they were not relevant. Reflecting this motivation, the American lawyer (later Supreme Court Judge) Ruth Bader Ginsburg (1975) wrote, “For impressionable minds, the word ‘sex’ may conjure up improper images” and so “the denotation and connotations of the word ‘gender’ make it more appropriate than ‘sex’” (p. 1).

This language shift to refer to sex using “gender” occurred alongside the already-mentioned feminist challenge to the socially constructed constraints upon women’s lives. (Duberman, 1975; Oakley, 2015). The term “gender” was also settled upon to describe the socially constructed expectations placed on people of each sex and their impact (Haig, 2004).

Finally, as described, “gender identity” was conceptualized in the 1960s and has gradually grown in cultural salience and prominence. It is often abbreviated to “gender” (Gribble et al., 2025; Sullivan et al., 2025). Thus, “gender” is being used to apply to the interrelated but distinctly different concepts of sex, the social expectations of the sexes, and gender identity.

## Confusion from Multiple Uses of Gender

The multiple meanings of “gender” make for difficulties in communication and comprehension. For example, in a recent paper on the importance of “sex and gender in research,” Ritz and Greaves (2024) used “gender” with at least two meanings. They described “gender” as a categorization, noting that people may be asked what their “gender” is, so seemingly using “gender” to mean “gender identity.” However, they additionally described “gender” as influencing the culturally supported “roles, norms, relations and opportunities” that people have, including how they dress and how children play, thereby clearly using “gender” to refer also to the social expectations of the sexes. The simultaneous use of different concepts of “gender” makes it difficult to perceive their meaning, let alone apply or respond to their arguments.

Lack of clarity through use of “gender” has enabled shifts in meaning of important concepts without it being clear this has occurred and without the implications of these shifts being properly discussed or considered in a type of “bait and switch.” For example, the term “gender equality” was coined to conceptualize equality between the sexes. It was thus operationalized as advocacy and initiatives toward achieving equality for girls and women with a clear understanding that this was necessary due to discrimination against female people. However, as the concept of gender identity has been institutionalized, gender equality is increasingly interpreted to mean equality between people of different gender identities (e.g., Government of Queensland, 2018). This change in meaning has not been subject to open discussion but has serious implications. For example, it means that gender equality initiatives which are understood as seeking to improve the situation of women may also include males. In a manifestation of this, the Scottish Government sought to legislate for public boards to include 50% women but understood “women” to mean people with a gender identity of woman rather than people of the female sex (Murray Blackburn Mackenzie, 2020). With this meaning, a 100% male board could be considered “gender equal” so long as half of the males had a gender identity of “woman.” In another example, a recent report from UN Women changed the meaning of “gender based violence” from referring violence against women to being violence to punish those defying “gender norms” and “norms of masculinity/ femininity” (Fuentes & Zulver, 2025). The latter is a very different meaning from the original; it excludes women and girls who do not challenge sex stereotypes but who nonetheless may experience violence because they are female while also including some males. Perversely, through such meaning shifts women who have advocated for the rights of girls and women as a sex class have been criticized as being “anti-gender equality” (Hines, 2025).

In another example, use of “gender” in UK government surveys assisted in the replacement of data collection on sex with data collection on gender identity (Sullivan et al., 2025). Prior to 1969, UK government surveys collecting data on sex used the term “sex” in their questions but from 1970, sex data were increasingly collected using the term “gender.” From 2010, there was a continuation of questions on “gender” but rather than collecting data on sex, these surveys were increasingly collecting data on gender identity with individuals given the ability to indicate that they were “non-binary” or “I identify another way.” Through this process, data collection on sex in the UK has declined with significant implications for the female data gap as well as crime and other statistics where sex is important (Gribble et al., 2025; Munzer et al., 2025; Sullivan et al., 2025). This change was enabled in a manner almost imperceptible to individuals and organizations due to the use of “gender” to denote both sex and gender identity.

Within law and policy, lack of clarity on whether sex or gender identity is meant can be particularly impactful. For example, in Australia, multiple legal proceedings have been undertaken to determine if legislation implementing the explicitly sex-based United Nations Convention on the Elimination of all forms of Discrimination Against Women defines women to mean those of the female sex or those with a gender identity of “woman” (Dumas, 2024; Gerber, 2024). The ultimate answer to this question will establish whether it is legal to discriminate on the basis of sex for purposes such as single-sex spaces for women, lesbian associations, or female sex-specific employment (such as rape crisis counsellors), or whether males with a gender identity of woman must also be included. Thus far, legal appeals have indicated that the latter is the case (Administrative Review Tribunal of Australia, 2025; Federal Court of Australia, 2024), and applications to allow for “female only” equality measures are only approved by the Australian Human Rights Commission if they are framed in terms of “female-identifying” people (e.g., Australian Human Rights Commission, 2023).

## Improved Terminology for Sex, the Social Expectations of the Sexes and Gender Identity

To facilitate clear communication, terms that clearly distinguish between sex, the social expectations of the sexes, and gender identity should be used. Here follows proposed terminology to improve clarity in relation to these concepts.

First, when referring to the reproductive categorizations of male and female, the term “sex” should be used and “gender” avoided. Furthermore, the scientific terms for the sexes of “male” and “female” should not be used to describe gender identities. This is particularly important in data collection where it is likely that many respondents will interpret questions with the answers of “male” and “female” as asking for their sex when the inquiry is intended to be in relation to gender identity (Balarajan et al., 2011). Accurate data collection on sex can be promoted by asking individuals to state their “sex as recorded at birth” or “birth sex.” Where it is necessary to define sex (e.g., in scientific, legal, or medical settings), the description should be in relation to sex as a reproductive classification rather than sex characteristics.

Second, when referring to the expectations that societies and cultures place on boys and girls, men and women and the resultant impact of this, the descriptors “social expectations of the sexes” or “social construction of the sexes” should be used. It has recently become common when discussing the interaction between sex and the social expectations of the sexes for the conjunction “sex/gender” to be used (e.g., Galinsky et al., 2024) but this should also be avoided due to the risk of confusion or conflation of the social expectations of the sexes and gender identity.

Third, when referring to gender identity, the term “gender identity” rather than “gender” should be used as the abbreviation risks confusion with sex. In circumstances where it is necessary to discuss issues related to individuals who have changed their legal sex, the term “certificated sex” or “legal sex” may be appropriate (United Kingdom Supreme Court, 2025). Table 1 outlines recommended terminology and terms to be avoided in relation to sex, the social expectations of the sexes, and gender identity and related concepts. This table is not exhaustive, and there will be additional concepts for which it is advisable that alternative terms be developed.

**Table 1:** Proposed terminology and terminology to be avoided in relation to sex, the social expectations of the sexes, and gender identity

Core concepts	Recommended terminology	Terminology to be avoided
The reproductive classifications of male and female	Sex	Gender
Social expectations of the sexes	Social expectations of the sexes Social construction of the sexes Socially constructed expectations of the sexes	Gender Gender norms
Gender identity	Gender identity	Gender

Concepts related to sex or the social expectations of the sexes	Recommended terminology	Terminology to be avoided
Equality between the sexes	Sex equality Sex equal Equality between the sexes	Gender equality Gender equal
Inequality between the sexes	Sex inequality Inequality between the sexes Sexism	Gender inequality Inequality between the genders
Equal outcomes for males and females	Sex equity	Gender equity
Measured differences between males and females	Sex differences	Gender differences
Difference in average earnings between men and women	Sex pay gap Male–female pay gap	Gender pay gap
Inadequate data collection and knowledge of women and girls’ bodies, physiology, health conditions, experiences and needs	Female data gap	Gender data gap
Evaluation of the different impact of spending and resource allocation on women and men	Sex equality budgeting Women’s equality budgeting	Gender budgeting
Ensuring that that the differences in needs and situation between the sexes are considered in the design, implementation and evaluation of policies, programs and projects so that inequality between the sexes is reduced	Sex equality mainstreaming	Gender mainstreaming
Measures to ensure that a certain percentage of positions are held by women	Women’s quota	Gender quota
Violence against women and girls rooted in sex inequality or the social expectations of the sexes. This may be violence perpetrated by men toward women and girls or may be undertaken by women (e.g., female genital mutilation)	Sex-based violence	Gender-based violence
Ideas about how someone should appear, behave or what they should be interested in or good at based on their sex	Sex stereotypes	Gender stereotypes
Non-conforming to stereotypes regarding how girls/women, boys/men should appear, behave or what they should be interested in or good at	Non-conforming to sex stereotypes Non-conforming to the social expectations of the sexes	Gender non-conforming
Sports or sporting activities in which physical differences between the sexes result in a sex-based performance disparity	Sex-affected sport	Gender-affected sport
Screening or testing to ensure those participating in women’s sport are female	Sex screening Sex testing	Gender screening Gender testing
Facilities or activities for both males and females	Sex-neutral Mixed sex	Gender neutral Unisex
Language which makes sex visible (e.g., women or mother versus person or parent)	Sexed language Sex-specific language Sex-based language	Gendered language

## Other Issues Related to Terminology

The words “women” and “men” (and indeed “girls” and “boys,” “mothers” and “fathers”) can be used to refer to an individual’s sex or their gender identity (Gribble et al., 2022). Defining terms via phrases such as “we use the words women and men in their sexed meaning of adult female and adult male people respectively” or “we use the words women and men to refer to the gender identities of individuals” may be necessary in some circumstances to ensure clarity of meaning.

Although outside the scope of this paper to address in detail, it is worth considering how the terms “patriarchy” and “misogyny” are commonly being misused. For example, rather than misogyny being understood as being hatred or prejudice toward girls and women, it may be used to mean hatred or prejudice toward femininity (Currah, 2024). In this meaning, actions toward equality for women, such as single-sex spaces, may be positioned as misogynistic because they exclude males who express themselves in a feminine manner (Colliver, 2021). Similarly, while “patriarchy” was conceptualized as a system and/or society organized in such a way that men hold power over women, it is now not uncommonly used to refer to structures oppressing any marginalized group (Pezaro et al., 2024). A reclaiming of the meaning of these terms or, at the least, providing definitions when they are used may be necessary.

In addition, care is needed when discussing the attributes, behaviors, and roles that are considered typical for boys and girls, men and women, that is “masculinity” and “femininity.” Some describe these as “maleness” or “femaleness” (Hoffman et al., 2005) allowing for reasonable inference that it is possible to be more or less male or female dependent on behavior, appearance, or personal preferences. Similarly, referring to “male” and “female” brains (e.g., Zhang et al., 2021b) or the “maleness” and “femaleness” of brains (e.g., Floris et al., 2018) falsely attributes sexes to a body part rather than whole individuals or further implies that sex is on a spectrum rather than recognizing the fact of variance between the sexes.

Finally, there is confusion caused by the terminology used to refer to transgender individuals. In the general population, a significant proportion of people may misunderstand the sex of a trans-woman to be female and of a trans-man to be male (Murray Blackburn Mackenzie, 2023). This complicates policy discussion but also places individuals at risk in health settings. For example, the delay of identification of pregnancy and life-threatening pre-eclampsia in a trans-man was seemingly contributed to by lack of recognition that “transgender men” are female (Stroumsa et al., 2019). The terms “male-to-female” and “female-to-male” to describe those who have undertaken medical treatments to appear as the opposite sex and the terms “sex reassignment” or “sex change” are also inadvisable. These descriptors may mislead people as to their ability to change their sex with potential for adverse outcomes such as where this results in them changing their sex marker in their health record.

## Conclusion

The use of the word “gender” to refer to sex, the social expectations of the sexes, and gender identity has and is hampering important discussions in relation to health and social policy and care with real-world implications. It is time for “gender” to be reserved for use only in relation to “gender identity.” Alternative terms for concepts related to sex and the social expectations of the sexes, as proposed here, are a starting point for improved clarity.
